# The Effect of Self-Compassion on Psychosocial and Clinical Outcomes in Patients With Medical Conditions: A Systematic Review

**DOI:** 10.7759/cureus.10998

**Published:** 2020-10-17

**Authors:** Ishita Misurya, Pranati Misurya, Anirban Dutta

**Affiliations:** 1 Department of Medicine, All India Institute of Medical Sciences, Jodhpur, IND; 2 Psychology, University of Delhi, New Delhi, IND; 3 Department of Medicine, North Eastern Indira Gandhi Regional Institute of Health and Medical Sciences, Shillong, IND

**Keywords:** positivepsychology, psychology, self-compassion, psychosocial, clinical, medical disease

## Abstract

Studies about the role of self-compassion have focused primarily on psychological well-being, but there is solid evidence to suggest that self-compassion may have larger and more prominent implications in the medical world. Therefore, this systemic review aimed to investigate the effects of self-compassion on psychosocial and clinical outcomes in medically ill patients.

A comprehensive search of several databases from their inception to August 10, 2020, was conducted, which included Ovid MEDLINE(R) and Epub Ahead of Print, Ovid Embase, Ovid Cochrane Central Register of Controlled Trials, and Cumulative Index to Nursing and Allied Health Literature (CINAHL). Eligible studies needed to include psychosocial or clinical outcomes of self-compassion in medically ill patients.

Nineteen articles (n=2,713 patients; 73.3% females) met our eligibility criteria and were included in this systematic review. There was a negative correlation between self-compassion and psychosocial outcomes such as anxiety, depression, and stress. Moreover, based on self-compassion intervention, there was an improvement in clinical outcomes related to diabetes such as hemoglobin A1c (HbA1c) and blood glucose levels.

This systematic review highlights the effect of self-compassion on psychosocial and clinical outcomes. Further studies are needed to evaluate long-term outcomes of a self-compassion-based-intervention to highlight its importance in the role of disease management.

## Introduction and background

Individuals suffering from medical illnesses are affected on both physical and psychological levels. The combination of exhaustion, pain, and decreased quality of life can cause self-doubt, low self-esteem as well as an inability to complete any personal or work-related tasks [1]. Psychological symptomatology of stress, anxiety, and depression can further exacerbate existing illnesses, contributing to a decreased desire for engaging in health-promoting or disease management behaviors [2-4]. Moreover, this burden can cause further psychological damage by causing individuals to direct blame at themselves for not being able to meet expectations of managing their illness [5].

In the past decade, evidence has emerged to suggest that self-compassion can be an important tool to help manage psychological issues. The concept of self-compassion, as defined by Neff, constitutes “being touched by and open to one's own suffering, not avoiding or disconnecting from it, generating the desire to alleviate one's suffering and to heal oneself with kindness [6]. Self-compassion thus involves offering nonjudgmental understanding to one's pain, inadequacies and failures, so that one's experience is understood as a part of the larger human experience” [6]. It involves the idea that individuals should treat themselves with the same care as they would treat their loved ones. The concept of self-compassion is further defined by three essential components, which include common humanity versus isolation, self-kindness versus self-judgment, and mindfulness versus over-identification [6].

Previous studies have demonstrated that self-compassion is linked to many factors of psychological well-being such as happiness, decreased anxiety, depression, stress, and a better quality of life [7-9]. A meta-analysis by MacBeth and Gumley revealed that individuals with high self-compassion reported having better mental health and quality of life compared to those with low self-compassion [10]. Furthermore, a study by Neff and McGehee demonstrated that self-compassion is correlated with resilience [11]. Another study by Neff, Kirkpatrick, and Rude demonstrated the protective role of self-compassion against anxiety [12].

To date, most of the research has directed its efforts toward the role of self-compassion in psychological well-being; however, there is evidence to suggest that self-compassion may well have a stronger contribution to make in the medical world as well. Although the research is limited to the role of self-compassion for health-related outcomes in medically ill patients, there are promising results to support the incorporation of self-compassion interventions to improve disease trajectory and management. This systematic review aimed to investigate the effects of self-compassion on psychosocial and clinical outcomes in medically ill patients.

## Review

Methods

Data Sources and Search Strategies

A comprehensive search of several databases from their inception to August 10, 2020, was conducted based on the Preferred Reporting Items for Systematic Reviews and Meta-analyses (PRISMA) guidelines [13]. The databases included Ovid MEDLINE(R) and Epub Ahead of Print, Ovid Embase, Ovid Cochrane Central Register of Controlled Trials, and Cumulative Index to Nursing and Allied Health Literature (CINAHL). The search strategy was designed and conducted by an experienced librarian. Controlled vocabulary supplemented with keywords was used to search for studies describing self-compassion and medical treatment. The actual strategy listing all search terms used and how they are combined is available in the Appendix section (Table 4).

Eligibility Criteria and Quality Assessment

Studies were deemed eligible if they met all of the following inclusion criteria: 1) investigate self-compassion; 2) involve patients aged more than 18 years with medical disorders, and 3) deal with psychosocial or clinical outcomes of self-compassion in medically ill patients. Case reports, conference abstracts and/or abstracts, and articles that were not in English were excluded from the study. The quality of each study was independently evaluated by two authors using the National Institutes of Health (NIH) Quality Assessment Tool [14]. Results of the quality assessment of all included studies are shown in the Appendix section (Table 5). All observational and cross-sectional studies were judged to be of good quality. The patients appeared to represent the whole experience of the investigator and the exposure and outcomes were adequately ascertained, and the length of follow-up was also deemed adequate.

Results

Study Selection and Characteristics

**Figure 1:**
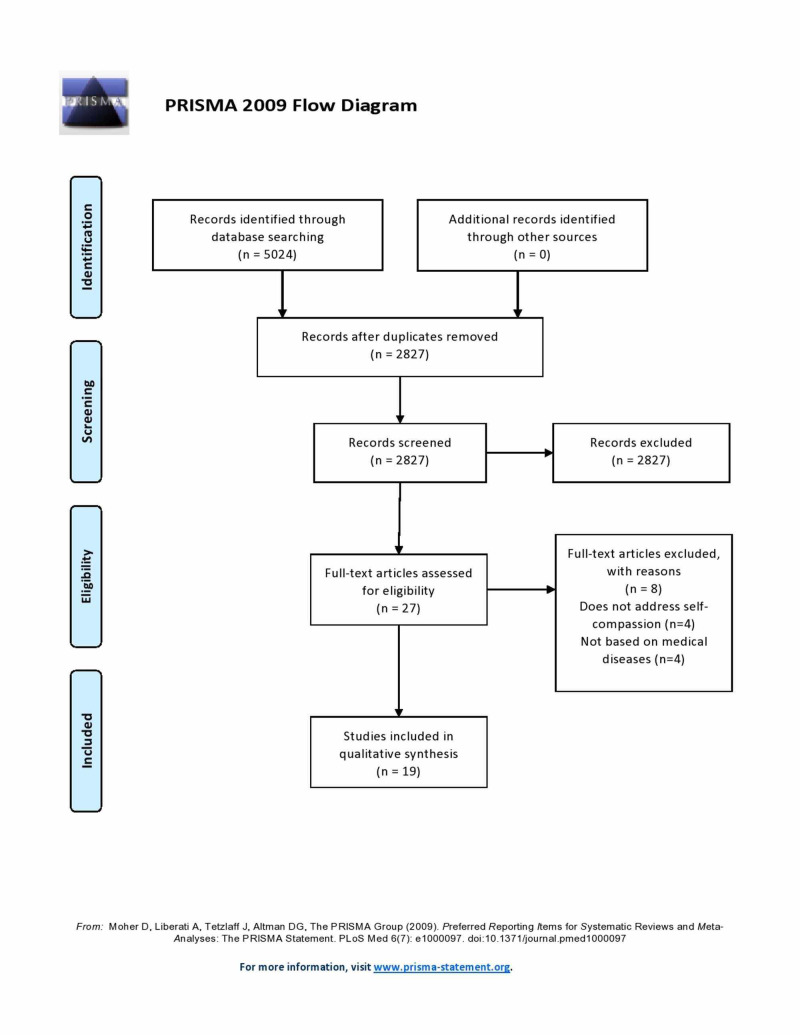
PRISMA 2009 Flow Diagram PRISMA: Preferred Reporting Items for Systematic Reviews and Meta-Analyses

A total of 5,024 records were identified from the initial search of electronic databases. After the exclusion of duplicated articles, 2,827 articles underwent title and abstract review. Following the exclusion of articles that did not fulfill the eligibility criteria, 27 articles underwent a full-length review. Eight articles were further excluded, for reasons shown in the Appendix section (Figure 1). Finally, 19 articles (n=2,713 patients, of which 73.3% were females) met our eligibility criteria and were included in this systematic review [15-33]. The baseline characteristics of the included studies are comprehensively described in Table 1.

**Table 1:** Baseline characteristics of included studies F: female; M: male; GCSE: general certificate of secondary education; NA: not applicable; T1DM: type 1 diabetes mellitus; T2DM: type 2 diabetes mellitus; TAFE: technical and further education; HIV: human immunodeficiency virus

Study	Year	Country	Sample size	Gender	Age (in years)	Education	Medical condition	Treatment of disease	Time from diagnosis	Study design
Abdollahi, Taheri, and Allen [15]	2020	Iran	210	All females	43.2 ± 7.4	Diploma (n=54); bachelor's degree (n=86); master's degree (n=28)	Breast cancer	Chemotherapy (n=155); radiation (n=147); hormone (n=164); reconstructive (n=73)	≤1 year (n=70); 1-2 years (n=61); 2-5 years (n=52); >5 years (n=27)	Cross-sectional study
Ambridge, Fleming, and Henshall [16]	2020	UK	66	32F, 34M	NA	GCSE (n=37); A-level (n=14); degree (n=10); doctoral degree (n=4)	Brain injury	NA	≤1 year (n=16); 1-2 years (n=15); 2-3 years (n=4); 3-4 years (n=4); 4-5 years (n=4); >5 years (n=21)	Mixed methods
Arambasic, Sherman, and Elder [17]	2019	Australia	82	All females	58.46 ± 8.77	Grade 12 or less (n=26); vocational (n=16); bachelor's degree (n=27); master's degree (n=13)	Breast cancer	Chemotherapy (n=53); radiation (n=59); hormone (n=55); targeted (n=15); reconstructive (n=21)	82.14 ± 19.34 months	Cross-sectional study
Brown et al. [18]	2019	UK	184	All females	51.54 ± 9.42	Degree and above (n=109); non-degree (n=71)	Breast cancer	Chemotherapy (n=116); radiation (n=135); hormone (n=139); targeted (n=30); mastectomy (n=57)	NA	Cross-sectional study
Dowd and Jung [19]	2017	Canada	220	202F, 17M, 1 preferred not to say	44.01 ± 13.33	NA	Celiac disease	NA	7.85 ± 7.85 years	Prospective study
Edwards et al. [20]	2019	USA	339	236F, 96M	51.66 ± 14.58	NA	Chronic pain	NA	NA	Cross-sectional study
Friis et al. [21]	2015	New Zealand	110	72F, 38M	47.6 ± 15.2	NA	T1DM (n=67); T2DM (n=20); type 2 insulin (n=23)	NA	16.7 ± 12.3 years	Cross-sectional study
Friis et al. [22]	2016	New Zealand	63	20M, 43F	44.37 ± 15.62	NA	T1DM (n=46); T2DM (n=9); type 2 insulin (n=8)	NA	16.84 ± 12.32 years	RCT
Hayter and Dorstyn [23]	2013	Australia	97	64F, 33M	40.1 ± 11.8	High school certificate (n=32); some high school (n=31); degree (n=15); postgraduate (n=6); apprenticeship (n=6)	Spina bifida	NA	NA	Cross-sectional study
Karami et al. [24]	2018	Iran	20	NA	Control: 43.57 ± 2.59; experimental: 44.38 ± 2.35	NA	T2DM	NA	NA	Quasi-experimental study
Klein et al. [25]	2020	USA	86	33F, 53M	29.7 ± 14.4	NA	Bleeding disorders	NA	NA	Cross-sectional study
Morrison et al. [26]	2019	UK	176	120M, 56F	64 ± 8	NA	T2DM	NA	12 ± 8 years	Cross-sectional study
Ferrari, Dal Cin, and Steele [27]	2017	Australia	310	58M, 252F	37.6 ± 15.1	High school (n=58); TAFE (n=81); university undergraduate (n=109); university graduate (n=35)	T1DM (n=203); T2DM (n=73)	Insulin injections (n=98); diet and exercise (n=97); insulin pump (n=76)	NA	Cross-sectional study
Hurwit, Yun, and Ebbeck [28]	2018	USA	259	41M, 218F	NA	NA	Multiple sclerosis	NA	NA	Cross-sectional study
Baillargeon et al. [29]	2018	Canada	48	All females	26.83 ± 5.98	NA	Vulvodynia	NA	NA	Cross-sectional study
Skelton et al. [30]	2020	USA	34	17F, 17M	47.79 ± 12.67	Grade 1-8 (n=2); some high school (n=7); diploma (n=11); some college (n=8); associates (n=4); college degree (n=2)	HIV	NA	18.77 ± 11.26 years	Cross-sectional study
Vasigh et al. [31]	2019	Iran	168	91M, 77F	43.13 ± 8.76	Literacy reading and writing (n=13); diploma (n=89); academic (n=66)	Migraine	NA	NA	Cross-sectional study
Wren et al. [32]	2012	USA	88	71.6%F	53.93 ± 9.65	Some high school or less (n=7); high school graduate (n=15); some college (n=35); college graduate or higher (n=31)	Musculoskeletal pain	NA	11.79 ± 10.23 years	Cross-sectional study
Zhu et al. [33]	2018	China	153	52M, 100F, 1 unknown	50.78 ± 11.61	Low (n=16); middle (n=103); high (n=30); missing (n=3)	Cancer	Chemotherapy (n=64); radiation (n=23); operation (n=22); Chinese medicine (n=12)	NA	Longitudinal study

Baseline Characteristics

As shown in Table 1, 19 studies were included of which four studies were from the UK, five studies from the USA, four studies from Australia, two studies from New Zealand, three studies from Iran, and one study from China; 2,713 patients were included of which 1,989 were female, with an age range of 26-64 years. Common medical conditions included were diabetes (n=5), breast cancer (n=3), multiple sclerosis (n=1), spina bifida (n=1), celiac disease (n=1), HIV (n=1), brain injury (n=1), migraine (n=1), musculoskeletal pain (n=1), and vulvodynia (n=1). Studies were mostly cross-sectional (n=14), followed by randomized controlled trials (n=2), mixed methods (n=1), longitudinal study (n=1), and quasi-experimental (n=1). Time from diagnosis ranged between 6-18 years for different medical conditions. Treatment options were included for breast cancer and diabetes. Breast cancer treatment options in different studies were similar and included chemotherapy, radiation, surgery, and hormone therapy. Treatment for diabetes included insulin pumps, insulin injections, and lifestyle modifications (diet and exercise).

Psychosocial Outcomes

Eighteen included studies exhibited outcomes of self-compassion using the Self-Compassion Scale (SCS) questionnaire as shown in Table 2. Five studies provided the values of self-compassion based on the average of all subscales from 1-5. The range for the self-compassion values was between 2.8-3.46. Three studies looked at specific subscales with the SCS [16,18,30]. A study by Ambridge, Fleming, and Henshall looked at the Self-Compassion Scale-Short-Form (SCS-SF), which was 5.69 ± 1.15 [16]. A study by Brown et al. demonstrated self-kindness: 2.74 ± 0.94, common humanity: 3.11 ± 0.93, mindfulness: 3.18 ± 0.83, and reflection: 1.70 ± 0.61 [18]. Lastly, a study by Skelton et al. observed scores of 64.12 ± 19.48 for compassionate engagement and action [30]. The rest of the studies reported SCS as an average of the total score, which ranged from 18-80.

Important Correlations 

All included studies evaluated the correlation of self-compassion with other important psychosocial outcomes such as depression, anxiety, stress, resilience, shame, quality of life, and other outcomes as shown in Table 2. Nine studies evaluated the correlation between self-compassion and depression [16,18,20-23,26,29,33]. All studies found that a higher self-compassion was correlated with lower levels of depression in individuals with a medical illness. Four of these studies looked specifically at self-compassion scores in relation to the Patient Health Questionnaire-9 (PHQ-9) scores, which found that increased SCS scores were associated with decreased levels of PHQ-9 scores [21,22,26,33]. Moreover, two studies looked at self-compassion in relation to the Hospital Anxiety and Depression Scale (HADS) questionnaire and demonstrated similar results [16,18]. Five studies looked at the correlation between self-compassion and anxiety, two of which were previously discussed using the HADS questionnaire. The rest of the three studies used different types of questionnaires but revealed that self-compassion scores were negatively correlated with anxiety [23,29,33].

Two studies looked at the relationship between self-compassion and shame [16,30]. One study showed that as self-compassion levels increased, shame decreased, while the other study showed no correlation between self-compassion and shame. Four studies looked at the correlation between self-compassion and quality of life [19,21,28,30]. Two studies showed that increased self-compassion improved quality of life, while two studies showed that higher levels of self-compassion correlate with any improvement [19,21,28,30]. Four studies investigated correlations between self-compassion and levels of stress [21-23,26]. Three of the four studies looked at self-compassion and Diabetes Distress Scores (DDS-17) and demonstrated that as self-compassion increased, DDS decreased [21,22,26]. The other study demonstrated that higher self-compassion levels correlated with lower stress levels [23]. One study by Hurwit, Yun, and Ebbeck demonstrated that higher self-compassion is associated with higher resilience [28]. Furthermore, the self-compassion interventions are likely to be more effective with women, as they have previously been reported to have lower self-compassion levels than men [6]. Lastly, two studies investigated the link between self-compassion and adherence behavior [19,30]. Skelton et al. demonstrated that self-compassion was not associated with increased adherence behavior in HIV patients [30]. On the other hand, Dowd and Jung exhibited that self-compassion at baseline was able to predict adherence to a gluten-free diet in celiac patients [19].

Clinical Outcomes

Only two studies investigated the effect of self-compassion on clinical outcomes specifically for diabetes with HbA1c and blood glucose levels, as shown in Table 3 [22,24]. Karami et al. demonstrated an improvement in blood glucose levels in patients who were in the intervention group (self-compassion program) compared to the control group at baseline and after the completion of the intervention [24]. The control group had a glucose level of 271 ± 35.88 at baseline compared to 272.75 ± 21.96 for the experimental group [24]. Post-intervention (after eight weeks), the control group had glucose levels of 267 ± 28.98 compared to 205.25 ± 12.55 for the experimental group [24]. Similarly, the other study by Friis et al. aimed to compare HbA1c levels between the control group and the experimental group [22]. They demonstrated that HbA1c levels improved after the intervention and at the three-month follow-up significantly in the experimental group (baseline: 74.25 ± 15.11; post-intervention: 71.44 ± 18.34; follow-up: 64.03 ± 16.25) compared to the control group (baseline: 64.04 ± 13.32; post-intervention: 66.03 ± 14.20; follow-up: 62.32 ± 12.41) [22].

**Table 2:** Effect of self-compassion on psychosocial outcomes BC Major Depression Inventory: British Columbia Major Depression Inventory; PHQ: Patient Health Questionnaire; SCS: Self-Compassion Scale, DDS17: 17-item Diabetes Distress Scale; CD-RISC 10: 10-item Connor Davidson Resilience Scale; QoL: quality of life; MSC: mindful self-compassion

Studies	Year	Questionnaires	Psychosocial outcomes	Correlations
Abdollahi, Taheri, and Allen [15]	2020	Self-Compassion Scale	Mean: 56.1 ± 9.25	Self-compassion moderates the relationship between perceived stress and self-care behaviors. Stress and self-compassion were significant predictors of self-care behaviors
		Perceived Stress Scale	Mean: 24.3 ± 5.23	
		Self-care utilization questionnaire	Mean 43.2 ± 7.4	
Ambridge, Fleming, and Henshall [16]	2020	Self-Compassion Scale-Short-Form	5.69 ± 1.15 (SCS-SF); 95.93 ± 6.01 (AQ)	A negative relationship at the .1 level (p = .055) was demonstrated between anxiety and self-compassion; participants who felt anxious were less likely to be self-compassionate. The results also illustrated a significant regression coefficient for self-compassion and shame (β = -1.615, SE = .515, t = -3.138, p = .003). This provides evidence that as the levels of self-compassion increased, shame decreased
		Self-awareness perceived responsibility	23.48 ± 32.89	
		Shame and guilt scale	11.51 ± 4.82 (shame)	
		Hospital Anxiety and Depression Scale	9.29 ± 5.24 (anxiety); 7.67 ± 4.09 (depression)	
Arambasic, Sherman, and Elder [17]	2019	General attachment style	Attachment avoidance: 3.12 ± 1.11; attachment anxiety: 2.51 ± 1.05	Both attachment anxiety and attachment avoidance negatively correlated with self-compassion
		Self-compassion	3.46 ± 0.57	
		Psychological adjustment (negative impact of cancer)	2.76 ± 0.77	
Brown et al. [18]	2019	Self-Compassion Scale	Self-kindness: 2.74 ± 0.94	Self-compassion subscales demonstrated a negative correlation with HADS, depression, and anxiety, although only smaller correlations were observed between common humanity than self‐kindness and mindfulness subscales. The final model included structural paths that showed that kindness and mindfulness scores, but not common humanity, uniquely predicted reduced brooding, depressive brooding, and worry
			Common humanity: 3.11 ± 0.93	
			Mindfulness: 3.18 ± 0.83	
			Reflection: 1.70 ± 0.61	
		Hospital Anxiety and Depression Scale	HADS anxiety: 8.33 ± 4.59	
		Ruminative Response Scale	HADS depression: 5.50 ± 4.01	
		Penn State Worry Scale	Worry: 3.19 ± 0.90	
Dowd and Jung [19]	2017	Celiac Dietary Adherence Test	Baseline: 11.78 ± 3.22; after: 11.18 ± 2.68	Both self-compassion and self-regulatory efficacy at time 1 had a direct relationship with the prediction of adherence to GFD at time 2. Self-compassion directly predicted celiac QoL
		Self-Compassion Scale	Baseline: 3.34 ± 0.75, After: 3.38 ± 0.76	
		Celiac QoL	After: 2.54 ± 0.80	
		Self-Regulatory Efficacy Scale	Baseline: 95.30 ± 8.85; after: 95.90 ± 8.94	
Edwards et al. [20]	2019	Self-Compassion Scale	75.90 ± 20.03	Self‐compassion accounted for a significant and unique amount of variance in physical and psychosocial disability, depression, pain acceptance, success in valued activities, use of traditional pain coping strategies, use of flexible pain coping strategies, and pain anxiety
		Sickness Impact Profile	0.24 ± 0.21 (physical)	
			0.25 ± 0.20 (psychosocial)	
		BC Major Depression Inventory	28.43 ± 16.33	
		Chronic pain questionnaire	47.40 ± 19.26	
		Pain Anxiety Symptom Scale	45.88 ± 22.22	
Friis et al. [21]	2015	PHQ-9	6.8 ± 5.6	As self-compassion increased, depression and diabetes-distress scores decreased. A positive relationship between distress and HbA1c among persons with lower self-compassion
		Diabetes Distress Scale	6.3 ± 2.7	
		Self-Compassion Scale	80 ± 16.8	
Friis et al. [22]	2016	Self-Compassion Scale	Pre-control SCS: 2.88 ± 0.60	Self-compassion increased in the MSC group between T1 and T2, with gains maintained at T3
			Post-control SCS: 3.12 ± 0.64	
			Pre-experiment SCS: 2.52 ±0.57	
			Post-experiment SCS: 3.10 ± 0.50	
		PHQ-9	Pre-control PHQ-9: 9.74 ± 6.06	The intervention reduced depression scores in the MSC group between T1 and T2, with results maintained at T3
			Post-control PHQ-9: 7.30 ± 5.02	
			Pre-experiment PHQ-9: 14.01 ± 4.52	
			Post-experiment PHQ-9: 9.16 ± 6.50	
		DDS17	Pre-control DDS17: 2.35 ± 0.63	
			Post-control DDS17: 2.29 ± 0.85	
			Pre-experiment DDS17: 3.16 ± 0.88	
			Post-experiment DDS17: 2.33 ± 0.86	
Hayter and Dorstyn [23]	2013	CD-RISC 10 scale	25.65 ± 8.07	Increased self-compassion was associated with lower levels of depression, anxiety, and stress
		Self-Compassion Scale	18.04 ± 3.90	
		Self-esteem	18 ± 5.43	
		Depression	11.92 ± 11.59	
		Anxiety	9.36 ± 9.39	
		Stress	13.90 ± 10.18	
Karami et al. [24]	2018	NA	NA	NA
Klein et al. [25]	2020	Self-Compassion Scale	3.45 ± 0.72	Hope and self-compassion together predicted physical and psychosocial QOL. Hope stood as the primary significant predictor of all three QOL scores
		Adult Hope Scale	53.3 ± 7.00	
		QoL	75.92 ± 16.62	
Morrison et al. [26]	2019	Self-Compassion Scale	3.29 ± 0.69	A negative correlation was found between levels of SCS and both depressive symptoms (PHQ-9) and diabetes distress (DDS-17)
		PHQ-9	5.4 ± 6.5	
		DDS-17	1.84 ± 0.90	
Ferrari, Dal Cin, and Steele [27]	2017	Self-Compassion Scale-Short-Form	NA	Self-compassion was correlated with higher well-being, lower HbA1c, higher self-management behaviors, and adherence to diet and exercise
		Diabetes Self-Management Questionnaire		
		Well-being questionnaire		
Hurwit, Yun, and Ebbeck [28]	2018	Resilience	3.60 ± 0.64	Self-compassion had a significant positive relationship with HRQoL and resilience. A similar relationship was found between resilience and HRQoL
		Self-Compassion Scale	3.32 ± 0.76	
		Health-related QoL	4.89 ± 1.24	
Baillargeon et al. [29]	2018	Self-Compassion Scale	2.81 ± 0.61	Women’s higher self-compassion was associated with their own lower anxiety and depression. Self-compassion was not associated with their own sexual distress
		Spielberger Trait Anxiety Scale	43.92 ± 9.60	
		Beck Depression Inventory-II	10.69 ± 6.25	
		Female Sexual Distress Scale	33.48 ± 9.83	
Skelton et al. [30]	2020	Self-Compassion Scale	64.12 ± 19.48 (compassionate engagement and action)	Self-compassion was not associated with adherence behavior, shame, or quality of life
		Experience of Shame Scale	49.71 ± 20.68	
		Adherence to Medication and Refill Scale	17.87 ± 5.67	
		QoL-HIV	40.30 ± 22.63	
Vasigh et al. [31]	2019	Mindfulness questionnaire	53.33 ± 4.69	There was no relationship between self-compassion and pain
		Self-Compassion Scale	71.48 ± 4.85	
		Numeric rating scale	3.33 ± 1.75	
Wren et al. [32]	2012	Self-Compassion Scale	19.37 ± 4.12	There was a correlation between self-compassion and pain self-efficacy, pain disability, negative, and positive affect
		Pain intensity	65.95 ± 26.16	
		Positive and Negative Affect scale	Positive: 2.79 ± 0.89	
			Negative: 1.75 ± 0.85	
		Pain Disability Index	35.89 ± 11.41	
		Pain Self-Efficacy Questionnaire	43.17 ± 16.42	
Zhu et al. [33]	2018	Self-Compassion Scale	39.7 ± 6.49	Self‐compassion total score at T1 was negatively associated with symptoms of depression, anxiety, and fatigue at T2
		PHQ-9	Baseline: 7.27 ± 5.74	
			After: 8.11 ± 6.47	
		Checklist Individual Strength	Baseline: 78.57 ± 23.88	
			After: 79.12 ± 20.81	
		State-Trait Anxiety Inventory	Baseline: 14.44 ± 4.14	
			After: 14.49 ± 3.89	

**Table 3:** Effect of self-compassion on clinical outcomes

Studies	Year	Parameter	Clinical outcomes
Karami et al. [24]	2018	Blood glucose levels	Pre-control: 271 ± 35.88
			Post-control: 267 ± 28.98
			Pre-experimental: 272.75 ± 21.96
			Post-experimental: 205.25 ± 12.55
Friis et al. [22]	2016	HbA1c (mmol/mol)	Pre-control: 64.04 ± 13.32
			Post-control: 66.03 ± 14.20
			Follow-up: 62.32 ± 12.41
			Pre-experimental: 74.25 ± 15.11
			Post-experimental: 71.44 ± 18.34
			Follow-up: 64.03 ± 16.25

Discussion

This systematic review included 19 studies involving 2,713 patients suffering from a variety of medical illnesses. The psychosocial outcomes of self-compassion scores were low and correlated with other parameters such as depression, anxiety, stress, shame, resilience, and quality of life. Moreover, two studies demonstrated the positive impact of incorporating a self-compassion-based intervention in disease management.

In previous studies, higher levels of self-compassion have been linked to lower levels of stress, depression, and anxiety [34,35]. For example, a group of healthy females was subjected to a high-stress task, and those who were trained in self-compassion were found to have lower sympathetic nervous system response compared to the placebo group [36]. Similarly, in another study that investigated the relationship between self-compassion and depression in a German cohort of 2,404 healthy individuals, Korner and colleagues used the PHQ-9 questionnaire to determine depression symptoms and demonstrated that increased depression correlated with lower SCS total scores and subscores [37]. After a logistic regression was performed and variables were adjusted, the model showed that 23% of the variance in depression symptoms was explained by SCS [37]. Therefore, self-compassion was shown to be a protective factor against depression [37]. Self-compassion has been demonstrated to have great implications on psychological well-being in chronic diseases given the contribution of psychological parameters in the exacerbation of diseases.

Another important role of self-compassion in medical disease management is related to the increase in health-promoting behaviors. Self-management behaviors have long been a central component of symptom management and improving disease trajectory and outcomes in chronic conditions [38]. The recent 2019 novel coronavirus pandemic has affected all spheres of health. Mental health has been no exception and resultant cognitive distress, anxiety, and fear of being out in public have been reported [39]. Self-compassion may prove to be quite an effective tool in managing these.

Previous studies have also demonstrated an association between self-compassion and self-management behaviors [3,4]. A meta-analysis by Sirois pooled 3,252 individuals across 15 studies and found that higher self-compassion was positively correlated to better engagement in health-promoting behaviors for chronic diseases [40]. These behaviors included better stress management, adherence to medication, lifestyle modifications, and improved sleep quality. This was consistent with data in our review, which demonstrated that in one of the two studies, self-management behaviors increased with increasing self-compassion.

Self-compassion-based training and interventions have been linked to better clinical outcomes in individuals experiencing medical illnesses. These interventions include compassion-focused therapy (CFT) and compassionate mind training (CMT) [41]. Previous studies have demonstrated success in these targeted interventions in providing acceptance and care for oneself by practicing health-promoting behaviors [42]. A review by Leaviss and Uttley, which included 14 studies, demonstrated that CFT is an effective intervention particularly in individuals prone to high self-criticism [43]. Although limited data exist in the role of self-compassion in improving clinical outcomes, there have been promising outcomes in the effect of self-compassion therapy in the improvement of treatment of medical conditions [44,45]. As shown by two studies in this review, self-compassion intervention compared to placebo can truly affect clinical outcomes in diabetes parameters such as HbA1c and blood glucose levels [22,24].

In this review, the studies examined the effect of self-compassion on clinical diabetic outcomes in the short-term, for a period of three months. There is a growing body of evidence that self-compassion interventions need to be provided through multiple sessions for at least 12 weeks to yield any differences in both psychosocial and clinical outcomes [46]. A study by Philips and Hine underlined the importance of a multi-session self-compassion intervention to impact self-management behaviors, improve psychological outcomes, and enhance physical health [46]. Thus, combining a self-compassion intervention with multiple sessions for a duration of more than six months can enhance the magnitude of self-compassion in its influence on disease management of individuals with medical illnesses.

The introduction of self-compassionate intervention is only the start in this realm; however, the practice of self-compassion by healthcare workers is necessary to maximize the impact of such an intervention. Studies have demonstrated that workers in the healthcare industry can affect the behaviors of patients [47]. Therefore, it is crucial to foster a compassionate setting to promote better communication, understanding, and disease management in patients [48,49]. This ongoing training and support will cultivate an environment that will enhance the patients' sense of self-efficacy and compassion toward themselves and thus improve their attitude towards engaging in health-promoting behaviors [50].

This study has several limitations. Firstly, the study only included publications in the English language. Secondly, there was significant variability in data presentation between studies. For example, the questionnaires used for each study varied. Moreover, even though the same self-compassion questionnaire was used, each study had calculated the scores differently by removing various items from the questionnaire. As such, this hindered our ability to conduct a meta-analysis and grasp the extent of the effects of self-compassion on psychosocial and clinical outcomes. Finally, only two of the included studies reported the role of self-compassion intervention on clinical outcomes, thus limiting our ability to identify whether using a self-compassion program can affect clinical outcomes and disease trajectory in medically ill individuals.

## Conclusions

In conclusion, this systematic review highlights the role of self-compassion with respect to its correlation and effect on psychosocial outcomes. Moreover, albeit the small sample size, this study showed the significance of the integration of a self-compassion program in the management of medical illnesses. Therefore, there is a dire need for the use of self-compassion as a tool to tackle the treatment of diseases. Further studies are needed to evaluate long-term outcomes of a self-compassion-based intervention to highlight its importance in the role of disease management.

## Appendices

**Table 4:** Search strategy used CINAHL: Cumulative Index to Nursing and Allied Health Literature

Search strategy employed in our review				
S5	S3 NOT (S4 OR TI child* OR TI youth OR TI adolesc* OR TI teen*)	Expanders - apply equivalent subjects; search modes - boolean/phrase	Interface - EBSCOhost Research Databases; search screen - basic; search database - CINAHL with Full Text	472
S4	(MH "Child+") NOT (MH "Adult+")	Expanders - apply equivalent subjects; search modes - boolean/phrase	Interface - EBSCOhost Research Databases Search Screen - Basic Search Database - CINAHL with Full Text	503,536
S3	S1 AND S2 AND (TI(regulat* OR self-compassion*) OR AB(regulat* OR self-compassion*)) AND (therap* OR treat* OR heal OR healing OR health OR recover* OR restor* OR recuperat*)	Limiters - English Language; publication type - journal article; expanders - apply equivalent subjects; search modes - boolean/phrase	Interface - EBSCOhost Research Databases Search Screen - Basic Search Database - CINAHL with Full Text	570
S2	(MH "Disease Management+" OR MH "Disease+" OR MH "Psychosocial Aspects of Illness+" OR MH "Severity of Illness" OR MH "Attitude to Illness+" OR disease* OR illness* OR chronic* OR disorder* OR patient OR patients OR condition OR conditions OR MH "Sexually Transmitted Diseases+" OR MH "Immunologic Diseases+" OR MH "Endocrine Diseases+" OR (MH "Nutritional and Metabolic Diseases+" OR MH "Skin and Connective Tissue Diseases+" OR MH "Congenital, Hereditary, and Neonatal Diseases and Abnormalities+" OR MH "Hemic and Lymphatic Diseases+" OR MH "Cardiovascular Diseases+" OR MH "Female Urogenital Diseases and Pregnancy Complications+" OR MH "Male Urogenital Diseases+" OR MH "Eye Diseases+" OR MH "Nervous System Diseases+" OR MH "Otorhinolaryngologic Diseases+" OR MH "Respiratory Tract Diseases+" OR MH "Stomatognathic Diseases+" OR MH "Digestive System Diseases+" OR MH "Musculoskeletal Diseases+" OR MH "Neoplasms+" OR MH "Virus Diseases+" OR MH "Parasitic Diseases+" OR MH "Bacterial and Fungal Diseases+" OR MH "Symptoms and General Pathology+")	Expanders - apply equivalent subjects; search modes - boolean/phrase	Interface - EBSCOhost Research Databases Search Screen - Basic Search Database - CINAHL with Full Text	4,324,677
S1	(MH "Self Regulation+" or self-compassion*)	Expanders - apply equivalent subjects; search modes - boolean/phrase	Interface - EBSCOhost Research Databases Search Screen - Basic Search Database - CINAHL with Full Text	6,852

**Table 5:** NIH Quality Assessment Tool NIH: National Institutes of Health; NA: not applicable

Criterion	Abdollahi, Taheri, and Allen	Ambridge, Fleming, and Henshall	Arambasic, Sherman, and Elder	Brown et al.	Dowd and Jung	Edwards et al.	Friis et al.	Friis et al.	Hayter and Dorstyn	Karami et al.	Klein et al.	Morrison et al.	Ferrari, Dal Cin, Steele	Hurwit, Yun, and Ebbeck	Baillargeon et al.	Skelton et al.	Vasigh et al.	Wren et al.	Zhu et al.
1. Was the research question or objective in this paper clearly stated?	Yes	Yes	Yes	Yes	Yes	Yes	Yes	Yes	Yes	Yes	Yes	Yes	Yes	Yes	Yes	Yes	Yes	Yes	Yes
2. Was the study population clearly specified and defined?	Yes	Yes	Yes	Yes	Yes	Yes	Yes	Yes	Yes	Yes	Yes	Yes	Yes	Yes	Yes	Yes	Yes	Yes	Yes
3. Was the participation rate of eligible persons at least 50%?	Yes	Yes	Yes	Yes	Yes	Yes	Yes	Yes	Yes	Yes	Yes	Yes	Yes	Yes	Yes	Yes	Yes	Yes	Yes
4. Were all the subjects selected or recruited from the same or similar populations (including the same time period)? Were inclusion and exclusion criteria for being in the study prespecified and applied uniformly to all participants?	Yes	Yes	Yes	Yes	Yes	Yes	Yes	Yes	Yes	Yes	Yes	Yes	Yes	Yes	Yes	Yes	Yes	Yes	Yes
5. Was a sample size justification, power description, or variance and effect estimates provided?	No	No	No	No	No	No	No	No	No	No	No	No	No	No	No	No	No	No	No
6. For the analyses in this paper, were the exposure(s) of interest measured prior to the outcome(s) being measured?	NA	NA	NA	NA	NA	NA	NA	NA	NA	NA	NA	NA	NA	NA	NA	NA	NA	NA	NA
7. Was the timeframe sufficient so that one could reasonably expect to see an association between exposure and outcome if it existed?	NA	NA	NA	NA	NA	NA	NA	NA	NA	NA	NA	NA	NA	NA	NA	NA	NA	NA	NA
8. For exposures that can vary in amount or level, did the study examine different levels of the exposure as related to the outcome (e.g., categories of exposure, or exposure measured as continuous variable)?	No	No	No	No	No	No	No	No	No	No	No	No	No	No	No	No	No	No	No
9. Were the exposure measures (independent variables) clearly defined, valid, reliable, and implemented consistently across all study participants?	Yes	Yes	Yes	Yes	Yes	Yes	Yes	Yes	Yes	Yes	Yes	Yes	Yes	Yes	Yes	Yes	Yes	Yes	Yes
10. Was the exposure(s) assessed more than once over time?	No	No	No	No	Yes	No	No	Yes	No	Yes	No	No	No	No	No	No	No	No	Yes
11. Were the outcome measures (dependent variables) clearly defined, valid, reliable, and implemented consistently across all study participants?	Yes	Yes	Yes	Yes	Yes	Yes	Yes	Yes	Yes	Yes	Yes	Yes	Yes	Yes	Yes	Yes	Yes	Yes	Yes
12. Were the outcome assessors blinded to the exposure status of participants?	NA	NA	NA	NA	NA	NA	NA	NA	NA	NA	NA	NA	NA	NA	NA	NA	NA	NA	NA
13. Was loss to follow-up after baseline 20% or less?	Yes	Yes	Yes	Yes	Yes	Yes	Yes	Yes	Yes	Yes	Yes	Yes	Yes	Yes	Yes	Yes	Yes	Yes	Yes
14. Were key potential confounding variables measured and adjusted statistically for their impact on the relationship between exposure(s) and outcome(s)?	Yes	Yes	Yes	Yes	Yes	Yes	Yes	Yes	Yes	Yes	Yes	Yes	Yes	Yes	Yes	Yes	Yes	Yes	Yes
